# Migrantisation: a key concept

**DOI:** 10.1186/s40878-025-00497-1

**Published:** 2025-09-25

**Authors:** Katharine Charsley, Nicole Hoellerer

**Affiliations:** University of Bristol, Bristol, UK

**Keywords:** Migrantisation, Migrant/citizen binary, Critical migration studies, Reflexive migration studies, Categorical migrantisation, Analytical migrantisation, Demographic migrantisation, Experiential migrantisation, Family migration

## Abstract

Migrantisation has become a key concept among scholars attempting to de-naturalise and de-centre the migrant/citizen binary. It has, however, been used in a variety of ways that have not always been clearly delineated. In this paper we tease out the strands of development of the concept, distinguishing different usages of migrantisation, and propose a new terminology to clarify the ways in which the term can serve a variety of purposes at different analytical levels. In particular, we identify two separate strands in the literature: ‘demographic migrantisation’ and ‘categorical migrantisation’. The latter is most consequential for Migration Studies, and so forms the main focus of this article, along with two further contributions developing from the categorical migrantisation approach: ‘migrantisation as an analytical perspective’, and the recently introduced concept of ‘experiential migrantisation’. Our discussion foregrounds the multi-dimensional nature of categorical migrantisation, which takes place across multiple domains, and is intersectional and contextual in character. In creating a more systematic understanding of the concept of migrantisation, we hope to lay clearer foundations for future research.

## Introduction

The term ‘migrantisation’^1^ rarely appeared in academic writing on mobility before 2010, but its use has increased rapidly in the last decade, becoming a key concept among scholars attempting to de-naturalise and de-centre the migrant/citizen binary and reveal the often racialised othering processes intertwined in the construction of the category of ‘migrant’. It has, however, been used in a variety of ways that have not always been clearly delineated, has not often been systematically explored as a concept in itself, has sometimes been employed in multiple senses within a single publication, and is often folded into other conceptual approaches such as the ‘doing migration’ framework (Amelina, 2020). In this paper we tease out and trace the multiple strands of development of the concept, proposing new terminology to differentiate between its various usages. In particular, we identify two separate strands in the literature: ‘demographic migrantisation’ and ‘categorical migrantisation’. The latter is most consequential for Migration Studies, and so forms the main focus of this article, along with two further contributions that have developed from the categorical migrantisation approach, which we call ‘migrantisation as an analytical perspective’ and ‘experiential migrantisation’.

The key insight of the ‘categorical migrantisation’ literature is the constructed nature of the category of ‘migrant’. Mobility is a human universal, but only certain instances are categorised as migration, and only some (mobile) people as migrants (Anderson, 2019; Balibar, 1991; Iosifides, 2017; Scheel & Tazzioli, 2022). The labels ‘migrant’ and ‘migration’ present as based on movement, in a similar way that the concept of ‘race’ often presents on the surface as about biological difference, concealing other dynamics involved in its ascription^2^. When subjected to critical analysis, both are revealed as socio-cultural and politicised practices of categorisation and othering, rooted in relations of power including histories of coloniality (Tazzioli, 2021). We term this strand of analysis – exploring the mechanisms, contexts and consequences of these practice – ‘categorical migrantisation’.

The ‘migrantisation as an analytical perspective’ approach has been advocated in particular by Bridget Anderson^3^ (2019) - a key figure in the migrantisation literature - as a way of challenging the migrant/citizen binary by viewing the citizen through the lens of migration. As such, it finds kinship in Janine Dahinden’s ‘de-migranticising migration research’ agenda (Dahinden, 2016; Anderson & Dahinden, 2021). Not all migration scholars, however, are content to follow the logic of the recognition of categorical migrantisation this far (e.g., Anthias, 2023). We propose the concept of ‘experiential migrantisation’ (first coined in Charsley and Wray (2023), and developed further here in light of our wider discussion of categorical migrantisation) as an intermediary intervention, allowing key insights from categorical migrantisation and the ‘analytical perspective’ approach to be operationalised in empirical research and analysis, even for those not pursuing the kinds of wider paradigmatic shifts suggested by Anderson and Dahinden.

This paper therefore makes three contributions. First, we identify differing usages of ‘migrantisation’ in the social science literature, introducing a new set of terms to distinguish between them (demographic migrantisation, categorical migrantisation, migrantisation as an analytic perspective). Second, drawing together the work of various scholars we articulate a model of ‘categorical migrantisation’ as multi-dimensional, differentiated and intersectional, to create a more systematic foundation for future research. Third, we propose ‘experiential migrantisation’ as a useful recent addition to conceptual tools in this area.

By bringing more clarity to the field, we hope to pave the way for greater conceptual precision in this important area of research. Whilst our focus is on categorical migrantisation and related approaches, we also suggest the possibility of a fruitful cross-fertilisation, as work on ‘demographic migrantisation’ could find new analytical avenues through the critical insights of categorical migrantisation scholarship.

The conceptual work set out in this paper emerged in the context of research on post-Brexit (im)mobilities^4^. The utility of this example lies in its illustration of key aspects of migrantisation: the official reclassification of mobile EU citizens as migrants, against a background of differentiated migrantisation of EU nationalities in British popular discourse, and the introduction of a new population into a restrictive immigration regime with profound consequences both migrant *and* citizen partners and family members.

Our conceptual review of relevant literature cannot claim to be comprehensive, and not just because we focus primarily on the academic anglosphere. The nature of existing scholarship on migrantisation does not lend itself easily to systematic review, as the concept is often employed as part of broader arguments rather than as the focus of an article, book or chapter. This lacuna reinforces the need for our work in drawing out, synthesising and systematising conceptualisations of migrantisation, but also presents challenges for search methods, as the term itself may not appear in titles, abstracts or keywords. Hence Scopus and Web of Science yield only a handful of results for the keyword searches ‘migrantisation’ and ‘migrantization’, missing significant contributions. We relied instead on Google Scholar which identified a far greater volume of relevant literature, weeding out non-relevant results and compensating for its limitations^5^ through supplementary identification of sources from citations.

### Migrantisation: multiple meanings

In the earliest usage we located, Gottschalk (1979) explores rural-urban mobility, using migrantisation in the sense of being made to migrate or relocate. Hence he writes: “[f]orced migrantisation, the annual ‘contract’ call-in system, ensures an annual conveyer belt of millions between town and country”. This meaning is echoed in Anderson’s recent formulation of ‘migrantisation in practice’ (2019) for situations in which immigration regimes or other factors necessitate migration, such as when British participants in our current research leave the UK after being unable to obtain immigration status for an EU partner. Neither usage is, however, widespread in contemporary academic literature.

A much more substantial body of work uses migrantisation in a demographic sense of an increasing proportion of migrants in a population. The study of ‘workforce migrantisation’ is particularly prevalent, for example, in relation to care work:

Care is… increasingly becoming a ‘migrantised’ occupation, not only in the West, but all across the world. Migrantisation is here defined as the process of incorporating migrant workers into the formal and informal care workforces of a country… A sector is undergoing migrantisation when the share of migrant workers therein has been increasing over time (Safuta et al., 2022, p. 304).

This, which we term ‘demographic migrantisation’, was for many years the dominant usage, for example in Kilkey et al.’s (2010) observations on the increasing reliance in Europe on ‘migrant as opposed to home-state domestic workers’, or Mezzadri’s (2008) work on North Indian factory labour where 90% of workers were migrants from poorer regions. This usage of migrantisation in a demographic sense continues as a distinct body of literature, especially concerning labour force migrantisation (e.g., Safuta et al., 2022; Fiałkowska & Matuszczyk, 2021), but also the migrantisation of populations and public spaces (e.g., Tamimi Arab, 2013; Haase et al., 2020). In this work, the key point is an increasing preponderance of ‘migrants’ (generally not problematised as a category for analysis) as a proportion of a population, space or sector.

In recent years, however, a different understanding of migrantisation has emerged as part of the reflexive turn in migration studies, and critical migration studies’ interest in challenging dominant categories. From this perspective, Riedner and Hess (2024, p. 2709) argue:

It is not ‘migrants’ who should be the focus of critical (migration) studies but the processes by which some groups are classified, labelled and objectified as ‘migrants’, what we understand as a process of ‘migrantisation’.

This approach, that we term ‘categorical migrantisation’, recognises and challenges the classification of some but not *all* mobile people (and some who are not mobile) as migrants. This vein of scholarship started in the early 2010s (e.g., Sunier, 2010), accelerating from the middle of that decade (Tazzioli, 2014; Römhild, 2017; Petersen & Schramm, 2017; Fiedler et al., 2017; Leimgruber, 2019), and finds kinship in the ‘doing migration’ school (Amelina, 2020). In the later 2010s some scholars, particularly Dahinden (2016) and Anderson (2019) took these insights further to argue for the utility of what we call ‘migrantisation as an analytic perspective’: as a lens through which we should also view citizenship. Finally, the recent formulation of ‘experiential migration’ (Charsley & Wray, 2023) draws insights from this work to highlight experiential aspects of migrantisation impacting the lives of some people who are not themselves categorised as migrants.

### Categorical migrantisation

A flurry of recent publications ask, in various forms, the question: ‘who is a migrant?’ (e.g., Amelina, 2020; Tazzioli, 2021; Scheel & Tazzioli, 2022). The insight that “not all foreigners and not only foreigners” (Balibar, 1991, p. 221) are categorised as migrants is by no means new, and can be seen as part of a more general post-modern social science critique of categories (and Weberian ‘ideal types’, e.g., Collyer & de Hass, 2012), but was given renewed stimulus by the ‘New Keywords’ Collective, a collaborative project exploring “terms and concepts that fill-out the contemporary problematic of migration” (de Genova et al. 2016). In particular, the influence of work on ‘illegalisation’ (De Genova & Roy, 2020, which in turn draws on the conceptual development of ‘racialisation’, e.g., Murji & Solomos, 2005) is clear both in citations and in the echo of the ‘-isation’ suffix. Borders, this work reveals, make migrants (De Genova 2015, p. 4). Anderson (2019, p. 7) crystalises the development of the concept we term ‘categorical migrantisation’ in this way:

What then are migration scholars and activists to do with the category of ‘migrant’?… Migration studies has got half-way to a successor concept in the debates about the terminology of illegality/undocumented/unauthorised/clandestine etc. No one is illegal, any more than they are witches, but they are ‘illegalised’ through an active social – and state endorsed – process. Perhaps then we can think of ‘migrantised people’ – though I would be grateful if someone could think of a less ugly word.

Drawing on the work of Sharma (2020), who traces the imperial routes of the category of ‘migrant’ and its underpinning of the nationalisation of states, Anderson argues for the importance of a focus on categorical migrantisation, on the grounds that the creation of the category of migrant has been key to the existence and function of nation-states:

[T]he form of the nation state is such that the labelling of some people as ‘nationals’ and others as ‘non-nationals’ or ‘migrants’ is not contingent but necessary. Nation states can make many different and varied kinds of populations… but they must make ‘migrants’ if they are to be a nation state (Sharma, 2020 in Dahinden and Anderson, 2021, p. 36).

In other words, “immigration and citizenship controls become crucial technologies for nation-making (and nation-maintaining) strategies” (Sharma, 2020, p. 3). The categories of ‘migrant’ and ‘migration’ are thus bound up with political power; “tied to the nation-state and the power it exerts over territory” (Collyer & de Haas, 2012, p. 470, cf. Wimmer & Glick Schiller, 2002). From a Foucauldian perspective, then, ‘migrants’ are part of the reality created by this power, transmitted and reproduced socially and politically through migrantising discourses.

‘Migrant’ is, however, a “highly heterogeneous status” with “gradations of outsider-ness” (Anderson, 2024). In the following sections, we suggest ways of systematising our understanding of this heterogeny, through a model of categorial migrantisation as processual, contextual, multi-domain and intersectional in nature. First, however, we trace the intellectual antecedents of the study of categorical migrantisation.

#### Intellectual antecedents

The extent to which those writing on categorical migrantisation cite the theoretical foundations for their approaches (beyond illegalisation and racialisation scholarship) varies, and authors frame their work in a variety of ways. For some, it is part of a de- or post-colonial project: deconstructing a category (migrant) with its roots in the logics and workings of Empire (Tazzioli, 2021; Sharma, 2020). Yildiz and Hill (2017, pp. 273-4) align their work with Said’s critique of the category and discourses of the ‘Orient’, and the post-colonial project of ‘reading against (the grain) – looking at social power relations from the perspective and experience of migration’. For Amelina (2020), exploring migrantisation is socio-constructivist, praxeological, and part of a wider sociology of knowledge and performativity, whilst others situate it within the recent reflexive and de-essentialising turn in migration studies (Anderson, 2019). These influences may combine, as when Worm (2023, p. 179) suggests that “migration is a specific sociopolitical category of governance and power, or doing difference, that emerged and became institutionalized especially in the Western centres of global hierarches from the late 19th century”^6^.

Part of the explanation for this variety of theoretical framings, beyond the intellectual groundings and preferences of individual writers, may be that the roots of identifying and analysing categorical migrantisation can be traced back much further, to some foundational elements of social analysis. We can, for example, see it as grounded in the labelling theory emerging with Durkheim (1897), and the crucial insight that labels are used by societies and bureaucratic bodies to categorise, manage and control ‘deviant’ individuals (cf. Zetter, 2007). We can also see the influence of Mead’s (1934) notion of self in bi-directional interactions with categories of others. Hacking’s (2007) model of ‘making up people’ develops this vein of thought in which institutions create, shape and utilise ‘new kinds of persons’ through bureaucratic labels.

We can also place the roots of analysis of categorial migrantisation in the longstanding concept of ‘othering’: ‘the organisation of cultural difference’ and distinction between ‘us’ and ‘them’, in which boundaries define social groups, not commonalities (Barth, 1998; Cohen, 1985; Bhabha 2012). This creation of similarity and difference is imagined, negotiated, contested and amended (Benedict Anderson 1982), but imagined differences have real world consequences – including the “potentially violent expulsion of those who are not ‘my blood, my family, my kin, my clan, my nation, my race’” (Voiscu 2013, p. 169), or even their physical destruction^7^. In this top-down process individuals may nevertheless have the ability to challenge their categorisation (also see discussions of victimisation and ‘spoiled identities’, e.g., Malkki, 1997).

The analysis of categorical migrantisation can therefore be situated within longstanding traditions of social analysis so foundational that authors may not consider it necessary to state that they are writing within these paradigms.

#### Categorical migrantisation: a contextual and intersectional approach

Whether or not a population or individual is categorised as ‘migrant’ is highly contextual, can change over time, and must be understood as related to intersectional positionalities embedded in relations of power (particularly to degrees of ‘privilege’ or marginalisation). Brexit provides ample illustration of these processes. While EU citizens moving to other EU states were officially defined as undertaking ‘mobility’ rather than migration, since Brexit, EU citizens moving to the UK and vice versa were (again) classified as migrants, appearing alongside other nationalities in immigration statistics:

The transformation of their status (and corresponding rights and entitlements) turned British citizens in the EU and EU citizens in the UK into ‘migrants’ and their movement into ‘migration’ (Benson et al., 2022).

Evidently, some people are migrantised without moving across borders, with borders instead moving across people^8^. Some of those supporting vulnerabl(ised) EU citizens in the UK fear a ‘new Windrush’ of insecurity (Parker et al., 2025), referring to the earlier scandal in which people moving legally from (former) British colonies to the UK were (re)migrantised several decades later through changes in citizenship and immigration status, followed by the intensification of border practices and rise of internal bordering (cf. Zehfuss, 2024). We also see what we might call the ‘re-migrantisation’ of some intra-EU mobile citizens through increasingly exclusionary welfare regimes (Riedner & Hess, 2024; Benson et al., 2022). Migrantisation is thus also an inherently contextual and temporal process (Zehfuss, 2024). Not only are histories of movement and current mobility interpreted to diagnose and ascribe migrant status, but suspicion of future border crossing can lead whole populations to be projected as potential future migrants, as in discourses about Turkey joining the EU (and therefore gaining migration rights to the UK) in the ‘Vote Leave’ Brexit campaign.

Anderson (2019, p. 8) neatly observes the intimate connections between racialisation and migrantisation: “once migration is no longer at the border it becomes ‘race’, and minority ethnic citizens are often already ‘migrantised’… who sheds and who retains their migrancy is often bound up with nationally specific ways of encoding and remaking of race”. Some racialised minorities who have not themselves moved internationally are habitually migrantised in public discourses. The term ‘second generation immigrants’ (and associated integration paradigms) is usually only applied to racialized ethnic minorities with family histories of international relocation (e.g., El-Tayeb, 2016; Chamberlain, 2023). As Tazzioli observes, for such people it is their presence rather than (actual) mobility that is problematised and presented as ‘other’ to the imagined nation, so that they are held in a “perpetual state of arrival” (Boersma & Schinkel 2018 in Scheel & Tazzioli, 2022), or as “space invaders” in Puwar’s memorable terminology (2004).

Migrantisation interacts with racialised hierarchies of deservingness, mobility and bordering (Tazzioli, 2021). The construction of migrants and of racial others share a history in the working of (post)colonialism and are intertwined in contemporary contexts (Dahinden & Anderson, 2021; Nowicka & Wojnicka, 2023; Scheel & Tazzioli, 2022). In some accounts, migrantisation appears as a subset or mechanism of racialisation – “the racialization of some individuals as ‘migrants’” (Tazzioli, 2021) - but more commonly the two have been conceptualised in the literature as separate but interacting (Scheel & Tazzioli, 2022). Indeed, writing in the context of Brexit, Tudor (2018, 2023) has argued for the utility of concepts of ‘migratism’ and ‘migratisation’ (which we and others phrase as ‘migrantisation’) to show the “power relation that ascribes migration to certain people” (2023, p. 230) as similar to and interdependent with, but separate from, ‘racism’ and ‘racialisation’. Migratism, Tudor (2023, p. 240) writes, “is not the same as racism, as not all ascriptions of migration are racist, but… racism very often functions through migratist strategies”. Race and migration may appear to collapse into each other in some contexts (Anderson, 2019), so “that the figure of the migrant has become a substitute for the biological notion of race in racist discourses and practices” (Scheel & Tazzioli, 2022, p. 8), but for analytical purposes one cannot be reduced to the other (Zehfus 2024).

Whilst the ‘discourses, categorizations, taxonomies and knowledge regimes’ of migrantisation are particularly entwined with processes of racialisation, they also have complex relationships with other dynamics of power (Scheel & Tazzioli, 2022). Some mobile people are not categorised as migrants, most obviously in the case of ‘expats’, where the interplay of nationality and class may lead to some individuals and communities escaping categorical migrantisation (Cranston, 2017; Kunz, 2016) - at least in their own and peers’ evaluations (we explore below the differing domains in which categorical migrantisation takes place). In popular discourses around EU migrants in the UK classed and racialised national identities, including the blurred boundaries of ‘whiteness’, also played into differentiated categorical migrantisation even before Brexit (e.g., Myslinska, 2024). (White) citizens of ‘old EU’ countries such as France and Germany were seldom described as ‘migrants’, in contrast to the frequent migrantisation of Eastern European arrivals (and racialised minority EU citizens). Others have explored how migrantisation can be related to gender, age or sexual orientation (Tudor, 2018; Scheel & Tazzioli, 2022). Categorical migrantisation is thus an inherently intersectional process shaped by and expressing multi-facetted and inter-related systems of power.

#### Beyond binaries: processes and degrees of categorical migrantisation

The category of migrant and its contrast in the figure of the citizen/native invites binary thinking – someone is or is not a ‘migrant’. Recognising such binaries as socially constructed is at the core of the various strands of social analysis set out above – from labelling theory and othering, to the post- and decolonial projects of ‘undoing and disengaging’ from longstanding state categories of difference (Sharma, 2020 in Tazzioli, 2021). Hence, Anderson calls for a critique of the “naturalisation of difference between migrants and citizens in the ‘migrant/citizen binary’ that structures not only public discourse but also much of (critical) migration research” (in Riedner & Hess, 2024, p. 2709).

Disengaging from and moving beyond binaries involves recognising the complexities, cross-cutting dynamics and contextuality of migrantisation. Tazzioli (2021, p. 385) poses the rhetorical questions: “is the binary opposition between ‘migrants’ and ‘natives’ sufficient to account for the degrees and hierarchies of racialized mobility? And how to register heterogenous bordering mechanisms not narrowed to national frontiers?”. Scheel and Tazzioli (2022, p. 11) suggest a processual, intersectional, multifaceted response:

[T]o study the processes of migrantisation at work… the discourses, categorizations, taxonomies and knowledge regimes they rely on, the processes of racialization they feature, their complex relationships to with class, age, gender, sexual orientation, their implications for those labelled and targeted as migrants and how the latter may try to negotiate, escape, defy or openly resist their migrantisation. By attending to these aspects, scholars will be able to show that processes of migrantisation are not only heterogeneous and contingent, but also relational and contested.

An important strand of recent writing emphasising the processual nature of categorical migrantisation seeks to analyse the processes, or ‘routines’ (Amelina, 2020), of migrantisation (Tazzioli, 2021; Worm, 2023; Riedner & Hess, 2024). Amidst this work, Amelina’s (2020) ‘doing migration’ approach is already gaining traction (e.g., Genova & Zontini, 2023; Worm, 2023). The terminology of ‘doing migration’, in addition to highlighting process, foregrounds performativity, echoing the earlier feminist formulation of ‘doing gender’ (West & Zimmerman, 1987).

What, then, is the relationship between ‘categorical migrantisation’ and ‘doing migration’? ‘Doing migration’ for Amelina (2020, p. 2) is synonymous with the ‘social production of migration’, referring to “all social practices that, being linked to specific categorisations and narratives of belonging, membership and deservingness (i.e. discursive knowledge), turn mobile (and often also immobile) individuals into ‘migrants’”. This appears to suggest that categorical migrantisation is only one element of broader practices of ‘doing migration’, but also that the total result of these practices is to turn individuals into ‘migrants’ – in other words, categorical migrantisation. The benefits of terming this ‘doing migration’ rather than ‘migrantisation’ are unclear. Moreover, whilst the ‘doing gender’ approach highlights individuals’ own performances of gender identities (in interactional, institutional contexts [West & Zimmerman, 1987, pp. 136-7]), ‘doing migration’ emphasises the role of external actors in discursively (and legally) producing the figure of the migrant. Migrantisation may not be an elegant term (Dahinden & Anderson, 2021), but in comparison to ‘doing migration’ it more intuitively communicates the turning of people into migrants, and mobility into migration. Whilst Amelina’s contribution to processual understandings of migrantisation is highly significant and we return to other aspects of her approach below, for now we treat it as aligned with and part of the analysis of categorical migrantisation.

#### Multiple domains

Categorical migrantisation is enacted through multiple processes. Here, we suggest the value of recognising these as existing in a variety of domains. Tazzioli (2021, p. 379) highlights the multiplicity of State processes: the “[l]aws, administrative measures, policies and public discourses [that] contribute to craft and define who is a migrant here and now, and to establish racialised hierarchies of (un)deservingness”. The State has the power to define people and populations as subject to immigration control, but migrants are discursively produced in multiple domains. These may coincide and reinforce each other to enact the same people or populations as ‘migrants’ in political discourses, statistics, and social interactions, but this cumulative model is only one possibility: processes in domains may also differ or be in tension. The categorical migrantisation of all EU citizens as subject to the UK’s immigration regime and enumerated in immigration statistics, for example, is not (yet) necessarily reflected in the construction of people from north-western Europe as ‘migrants’ in everyday interactions away from the border. We can therefore understand categorical migrantisation as operating in multiple domains which may or may not interact (cf. Spencer & Charsley, 2016), and in which different definitions and connotations of ‘migrant’ may operate.

Anderson (2019, p. 2) illustrates this complexity:

These three types of migrants do not easily map on to one another. For example, while the migrant in data is typically defined as foreign born, many of those ‘migrants’ may be citizens in law, through naturalisation for example, or deriving citizenship from a parent despite being born abroad. On the other hand, a person might be foreign born, and a non-citizen in law, but still not imagined as a ‘migrant’ in public debate. British people living abroad rarely think of themselves as ‘migrants’ and certainly not ‘illegal immigrants’ whatever their status in practice. They are expats. The middle class, wealthy and white may experience immigration controls as collateral damage, but they do not imagine themselves as the primary target of controls, and very often they are right. Putting it crudely, in political debate, a ‘migrant’ is a person whose movement, or whose presence, is considered a problem. In academic research different disciplines tend to select different definitions: demographers and economists tend to prefer the definition ‘foreign born’ because it does not change; lawyers, ‘foreign national’; while sociologists and anthropologists are often more concerned with the migrant as someone who is perceived/perceives themselves as out of place or racially othered.

Amelina (2020) uses the macro-meso-micro format to distinguish between institutional, organisational, and interactional levels of what she calls ‘doing migration’ and we term ‘categorical migrantisation’. At the institutional (macro) level are migration regimes and associated narratives. The organisational (meso) encompasses bordering, surveillance and other mechanisms of discipline. Interactional (micro) aspects include how routines of daily face-to-face interactions generate “microforms of ‘migration’ by stigmatisation, while also giving the potential to resist the social attribution as ‘migrant’” (Amelina, 2020, p. 1).

Combining this framework with a multiple domains model would allow for systematic and fine-grained analyses in which processes within and across each domain may also operate at various levels. In the legal domain, for example, post-Brexit EU migrants to the UK are (as newly categorised migrants) subject to the institutional immigration system (macro), which also entails new encounters with bordering mechanisms (meso), and face-to-face interactions with border staff and, for some who can afford it, immigration lawyers (micro).

Both the macro/meso/micro and domains model are, of course, heuristic devices rather than empirical differentiations. In migration studies more generally, there has been variation in which aspects are allocated to the ‘meso’ level in particular. Similarly, the selection and boundaries of domains for analysis varies:

- Anderson (2019) distinguishes between migrantisation in data, law and policy, academic discourse, self-perception and public debate.
- Riedner and Hess (2024, p. 2709) list “political, social, structural and knowledge-based practices of categorisation and othering”.
- Charsley and Wray (2023) also distinguish between the legal, political and social.
- Scheel and Tazzioli (2022, p. 11) make more fine-grained distinctions between “bureaucratic assessments, administrative processes and related dialogues of action”.
- Benson et al. (2022) add academic funding schemes as a further area in which instances of mobility may or may not be categorised as migration.

The variety of these articulations is instructive in itself, underscoring the pervasive nature of categorical migrantisation across diverse aspects of social life. Rather than attempt to compile or arbitrate a definitive schema of domains, we suggest the value of this approach lies in awareness of this multiplicity. The domains most relevant to a particular research project will vary, but this awareness demands that researchers consider and specify the domains addressed in their work (and acknowledge those not addressed – cf. Spencer & Charsley, 2016 on integration).

In practice, the focus might be on one domain, but interactions with other domains can be traced and acknowledged. Amelung et al. (2024, pp. 2168-9), for example, are particularly interested in data:

[F]rom an epistemic point of view, migration does not exist independently of the concepts, definitions, methods, statistics, visualisations and various other data practices that are mobilised to produce knowledge on migration for the purposes of its ‘management’ (Scheel et al. 2019)… Consequently, migration only becomes tangible as a debatable, actionable reality and object of government, as an effect of knowledge and data practices as well as related artefacts generated by these practices. These include, for example, charts, maps and tables presenting numbers on stocks and flows of migrants.

They illustrate this perspective in the changing categories for classifying German populations in relation to migration: from ‘person with migration background’ (by which measure a quarter of the population was migrantised, half of whom had citizenship, with many born and raised in Germany) to ‘people with immigration history’ (encompassing only international arrivals and their direct descendants – reducing the migrantised population by 10%). They point to the political implications of this reclassification, both in deflating the ‘integration problem’, but also in claims about diversity. These data practices therefore have implications in the political domain, as they “enact the very realities they are meant to elucidate, measure and describe” and so “shape and prefigure related problematisations and narratives in public debate and, eventually, policy interventions and practices of government” (Amelung et al., 2024, p. 2169).

#### Resisting defining the ‘migrant’

Before we move on from our consideration of categorical migrantisation, the question remains of the definition of ‘migrant’ implied within this form of migrantisation – beyond a broad sense of ‘outsiderness’ or ‘otherness’ presented as based on geographical mobility. Scheel and Tazzioli (2022) propose to shift the focus from the problematic statist classifications of ‘migrant’ to a migrant-centred perspective focussed on ‘border struggles’ (following Mezzadra & Neilson, 2013). A migrant, they suggest, is “a person who, in order to move to or stay in a desired place, has to struggle against bordering practices and processes of boundary-making that are implicated by the national order of things” (Scheel & Tazzioli, 2022, p. 3). This definition is intended to recognise the plurality and contextuality of struggles and migrant perspectives, but at the same time suggests that “not all people subjected to border controls of processes of boundary making are migrants… Only if people’s presence in or right to move to a desired place is denied or called into question because they are considered ‘as the others of Nation-Natives’ (Sharma, 2020, p. 13) these people will qualify as migrants” (Sheel and Tazzioli 2022, p. 10).

Whilst the purpose of an anti-essentialist, bottom-up definition recognising that ‘migrants’ are constructed in relation to borders is clear, the urge to (re)define ‘migrant’ sits somewhat awkwardly with the core constructivist insight that the ‘migrant’ does not exist empirically, and that the term should be understood as a problematic construction. It also leads Sheel and Tazzioli (2022) to grapple with the ‘liminal’ or ‘ambiguous’ cases that do not quite fit their definition. A ‘categorical migrantisation’ approach could, on the other hand, recognise the category of ‘migrant’ as an almost empty signifier that can take on a variety of meanings in different domains, and be used for different purposes in political and other discourses, albeit with common connotations of an individual or group whose mobility or whose presence in combination with attributed past mobility, is problematised. Indeed, as migrantised people may learn and appropriate categories applied to them (Drotbohm 2024), positive redefinitions of the term are possible. Hence, ‘migrants’ in our understanding, are simply those who are categorically migrantised, according to whatever definition and connotations of the term exist in the relevant domain-specific, geographical and temporal context. Like ‘race’ or ‘integration’, this ability to shift meaning is part of the power of the category of ‘migrant’.

### Migrantisation as an analytical perspective

Some scholars have built on the insights from the study of categorical migrantisation to suggest we can go further, to what we call ‘migrantisation as an analytical perspective’. Once again, there have been multiple variations of this approach, from the relatively straightforward viewing of phenomena with a focus on migration (cf. Falk’s [2019, p. 18] “migrantisation of the past”: “history systematically told from a perspective of migration”) to more complex calls for ‘migrantisation’ or ‘de-migranticisation’ of research, suggesting a more radical rethinking of migration studies itself (Dahinden and Anderson, 2021) – these latter perspectives are the focus of this section.

As we note above, the scholarship on categorical migrantisation already entails an analytical shift in de-naturalising the category of ‘migrant’, and exposing its foundations, processes and consequences. We see this in the cognate conceptual development of the ‘doing migration’ approach, which is set out as both a description of processes enacting the figure of the ‘migrant’, and an analytical approach focussed on these processes. For Amelina (2020, p. 3), the ‘doing migration’ approach is based on five conceptual premises:

1. The social production of migration starts with the categorisation as migrants/non-migrants.
2. These discursive processes performatively articulate or realise their subjects and objects.
3. Practices differentiating between migrants and non-migrants are ‘routines of doing migration’.
4. The concept of ‘migration is a result of these routines, grounded in ‘historically specific discursive knowledge’.
5. “The nexus between routinised practices of ‘doing migration’ and discursive knowledge… generate historically specific social orders of migration”.

Hence, Worm (2023, p. 180) takes the ‘doing migration’ approach to entail looking at the social practice of constructing some mobility as migration, with ‘migrantisation’ understood in a narrower sense of “labelling movement within migration regimes and in everyday life figurations”. For the reasons noted earlier, however, we prefer a broader understanding of the study of categorical migrantisation which encompasses exploration of its mechanisms across multiple domains. From this perspective, Amelina’s five conceptual foundations are a highly valuable conceptualisation of the mechanisms and consequences of categorical migrantisation.

Anderson and Dahinden (2021), however, argue for more wide-ranging analytical transformations, suggesting that migrantisation (and ‘demigranticisation’ in Dahinden’s longer formulation) can constitute a more paradigmatic shift in social science. These approaches can be seen as reflexive responses to the insights of categorical migrantisation, and part of a growing literature critiquing the often statist and dehumanising ontological bases of migration studies (Scheele & Tazzioli, 2022; Dahinden, 2016). But they also respond to other critical interventions: the early 2000s methodological nationalism critique (Wimmer & Glick Schiller, 2002); the mobilities perspective which sought to de-exceptionalise mobility, placing its many forms at the centre of understanding of society (Urry, 2007); and parallel debates in German sociology (Kiepenheuer-Drechsler, 2013; Bojadžijev & Römhild, 2014) brought into the academic anglosphere by De Genova et al. (2016) and Dahinden (2016).

In the late 2010s and early 2020s, these intellectual strands were developed further by Anderson and Dahinden – variously arguing for the ‘demigranticisation of migration studies’ or the ‘migranticization of social science’ (Dahinden, 2016), and ‘migrantisation of the citizen’ (Anderson, 2019). The key thrust of both approaches is to de-exceptionalise migration or mobility and analytically deconstruct the migrant/citizen binary. For Dahinden (2016, p. 2207), “migration and integration research originates in a historically institutionalized nation-state migration apparatus and is thus entangled with a particular normalization discourse. Therefore, this field of study contributes to reproducing the categories of this particular migration apparatus”. ’Demigranticizing’ (cf. Kiepenheuer-Drechsler, 2013) migration research entails denaturalising the category of migrant as the focus of study, so it is not automatically part of research questions and designs as a category of difference, and its relevance is analysed only in a second step (cf Brubaker, 2006 on ‘ethnicity without groups’).^9^

For Anderson on the other hand, migrantisation as an analytical perspective means viewing citizenship through the lens of migration to reveal differentiated rights, including ways in which citizens are impacted by immigration regimes. She calls this ‘migrantising the citizen’: “The instability of the category of ‘migrant’ after all destabilises the category of ‘citizen’” (2019, p. 8). In other words, she seeks to problematise the migrant/citizen binary: “to make connections between the formal exclusions of noncitizenship and the multiple, and sometimes informal exclusions within citizenship” (2019, p. 2), including those related to forms of mobility not labelled as migration.

Anderson sums up the relationship between the two analytical approaches in this way:

…we are attached to the field for different reasons. Dahinden’s primary interest is in knowing when being a migrant matters. Only once we have de-migrantised can we see the significance of migration both for the people themselves and for others. My primary interest is using ’migration’ as means to understand the conditions of marginalised populations more generally. The two are, in the final analysis, not possible to separate, and in fact we need both if we are to build on the insights of migration studies and move beyond them (in Dahinden and Anderson, 2021, p. 38).

It would, of course be possible to address such critiques through other lenses than ‘migrantisation’ - for example simply through the complexities of citizenship (cf. Anderson, 2013). But using the term ‘migrantisation’ in both empirical discussions of categorisation, and in the more radical ‘analytical perspective’ sense, with its call to rethink migration studies, is part of Anderson’s broader project of using the concept of ‘migrant’ as an intellectual lever in various contexts – we return to this below.

### Experiential migrantisation

In this final section we suggest the utility of an addition to the conceptual frameworks derived from the insights of categorical migrantisation: ‘experiential migrantisation’. This concept was originated by Charsley and Wray (2023) in the context of research on families impacted by the UK immigration regime. We expand on it here in light of the discussions of migrantisation above. Whilst this formulation responds to calls to ‘migrantise the citizen’ by highlighting ways in which citizens can experience aspects of migrantisation without being categorised as migrants, it does not necessarily require the adoption of the wider analytical shifts advocated by Anderson and Dahinden (or indeed recent calls to ‘dismantle’ Migration Studies – e.g., Vigneswara, 2025).

In studying categorical migrantisation we can distinguish between practical consequences (e.g., having to apply for a visa), and experiential aspects (e.g., impacts on senses of security and belonging). As Hacking (2007, p. 293) points out, those classified are then ‘not quite the same kind of people as before’. If migrantisation has consequences for the migrantised (Worm, 2023), an interest in categorical migrantisation includes how social actors talk about, interact and practice these categories - both in terms of (negative) experience, and possibilities for resistance (Dahinden & Anderson, 2021, pp. 36–37). How do individuals respond to their migrantisation (Worm, 2023)? How do they define their own (im)mobilities and link them to belonging?^10^ And how does the experience of being migrantised vary (Calum, 2022)? In other words, rather than only analysing an objective, etic perspective of migrantisation, the subjective, emic, insider perspectives of being migrantised are also analytically important.

For Toukolehto (2023), a key question is to what extent people develop a ‘migrant subjectivity’: a conscious construction of oneself as a ‘migrant’ in response to categorical migrantisation. Echoing Du Bois’ (1903) ‘double consciousness’ in which the subordinated also see themselves through the eyes of the other, this internalised ‘otherness’ has ‘repercussions for one’s sense of self, sense of belonging, sense of social positionality as well as one’s capacities for autonomy and agency in the context that produces such subjectivity’ (Toukolehto, 2023, p. 87).

Individuals may resist their migrantisation, or at least its consequences. Categorical migrantisation is ‘relational and contested’ (Scheel & Tazzioli, 2022), so those labelled and targeted as migrants may try to “negotiate, escape, defy or openly resist their migrantisation” (Scheel & Tazzioli, 2022, p. 11). In our work with UK-EU couples, for example, whilst some expressed a new sympathy for others subjected to immigration regimes, others employed dominant immigration discourses to argue that *their* migrant-ness was different from more commonly problematised irregular migrants, refugees, or those moving from ‘less developed’ countries, and so argue that *their* mobility should be regulated in more streamlined and forgiving ways.

Crucially, however, both UK and EU partners narrated these identities. Charsley and Wray (2023) observe that citizen partners can share experiential aspects of their non-national family members’ migrantisation. Drawing together categorical and analytical perspectives, they (Charsley & Wray, 2023, pp. 382-3) argue:

At the heart of family migration crises is a form of ‘migrantisation’, a concept that recognises the constructed nature of the category of ‘migrant’ as applying only to some types of mobile people**…** It can also describe the process by which immigration status may change… A second, related strand in the literature uses these insights to argue for a perspectival shift recognising the ways in which citizens are impacted by immigration regimes**…** Such migrantisation may be experiential in character, when citizens are subjected to an immigration regime they previously assumed to apply only to migrant ‘others’. For British citizens in relationships with foreign partners, the encounter with the immigration regime governing their right to live with their chosen family in their country of citizenship is a crisis of what we term ‘experiential migrantisation’.

In other words, citizens with non-citizen partners - although not themselves categorized as ‘migrants’ - become impacted by immigration regimes through their connection to a categorically migrantised partner (what Odasso, 2025 calls ‘migration of contact’). In mixed citizenship families, citizen and non-citizen members share experiences of threats to their family lives. Hence, British women with ‘deportable’ partners reported flinching at the sight of immigration enforcement, and keeping suitcases packed in case the family need to leave urgently (Griffiths, 2021). South Korean women married to racialized migrant men find themselves exposed to racism, social exclusion and othering (particularly when pregnant), as well as undermining their sense of national belonging, and may respond by developing transnational orientations and plans to move abroad (‘migrantisation in practice’) (Kwak, 2018). Similarly, Lyytinen (2024) reports Finnish women with deportable partners losing spatial-temporal control over their lives, feeling compelled to leave the country to maintain their relationship, whilst simultaneously unable to leave children (with ex-partners) in Finland.

Lyytinen is, however, ambiguous about the term ‘experiential migrantisation’ – finding it a useful tool for exploring the impact of deportability on mixed status families, but concerned over potential erasure of the particularity of experience of those classified as migrants. However, as we set out above, the experience of categorical migrantisation also varies by degree and domain – whilst some legally classified as migrants do not experience migrantisation in their social lives, others are subject to discrimination and deportability. Whilst citizens in relationships with migrantised partners may not be ‘deportable’, some share key experience of migrantisation: racialised discrimination, lack of control over their lives, mistrust and anger towards the authorities responsible for their experiential migrantisation, social rejection by families and friends, and forced separation from partners and/or children (Lyytinen, 2024; Charsley & Wray, 2023). Although ‘citizens’ and ‘migrants’ may experience administrative violence differently, the concept of ‘experiential migrantisation’ highlights these connections.

We explore experiential migrantisation in the narratives of participants in our current research more fully elsewhere (Hoellerer & Charsley, 2024), but some brief illustrative examples may be useful. British citizen participants often spoke not of the experience of their EU partners in the immigration system, but of their shared experience as a couple or family, with the state seen as standing in the way of their shared hopes of making a life together in the UK. Like categorical migrantisation, experiential migrantisation varies by domain, spatial-temporal context, and with intersectional positionalities. Some are easy to predict (socio-economic status, racialization, etc.), but their impacts are contextual and subject to change. Some British citizens’ ability to meet financial requirements to sponsor their partners reduced the severity and duration of their experiential migrantisation (although the process is often still lengthy, see Charsley & Wray, 2023), but a dramatic increase to the income requirements in 2024 brought many more British people into direct content with state violence against their imagined futures. Those unable to meet visa requirements nervously attempted to maintain their relationships through extended visits – aware that questioning at the border could bring an end to this possibility (Brexit Couples Project, 2024). ‘Experiential migrantisation’ therefore highlights how experiences of migrantisation may be shared across divisions of immigration status, between ‘migrants’ and ‘citizens’. The study of mixed citizenship families provides perhaps the most obvious arena to operationalize the ‘migrantising the citizen’ approach through ‘experiential migrantisation’, as an empirically-grounded step towards ‘demigranticising’ migration studies, by exploring impacts of immigration regimes beyond those classified as migrants.

## Concluding discussion

This article has sought to identify and disentangle the various ways in which ‘migrantisation’ has been used in the migration studies literature and suggests ways forward. We identify and name two main bodies of literature. Work on what we term ‘demographic migrantisation’ uses the term to denote contexts in which increasing proportions of a population are migrants (e.g., in the care sector workforce). The second explores ‘categorical migrantisation’: the classification of some mobile (and nonmobile) people as ‘migrants’, and the processes through which this classification operates and impacts on those subject to this classification. This strand of work has further-reaching significance for Migration Studies and formed the main focus of our discussion. Bringing together scholarship in this vein allowed us to set out a model of processes of categorical migrantisation as heterogenous, intersectional and contextual, and taking place across multiple domains. We then discussed two developments arising from the categorical migrantisation approach. ‘Migrantisation as an analytical perspective’ suggests a perspectival shift, challenging the migrant/citizen boundary and de-exceptionalising mobility. The recent formulation of ‘experiential migrantisation’ takes core insights from previous migrantisation scholarship (in its ‘categorical’ and ‘analytical perspective’ iterations), translating them into a conceptual tool highlighting ways in which citizens can also be profoundly impacted by migration regimes.

Why is it important to recognise these different usages? In Anderson’s work, after all, multiple connotations of ‘migrantisation’ are treated as a strength, and part of her intellectual project is to insert the categories of ‘migrant’ and ‘migrantisation’ at various levels of analysis to explore their utility and leverage. In the following passage, for example, she moves between ‘categorical migrantisation’, the ‘analytical perspective’ and ‘migrantisation in practice’ (being made to migrate):

Migrantising the citizen is precisely what the everyday terminology of ‘second generation’ migrant or ‘person of migration heritage’ encourages us to do. Who sheds and who retains their migrancy is often bound up with nationally specific ways of encoding and remaking of race… minority ethnic citizens are often already ‘migrantised’… Stevens (2011) for example has found that thousands of US citizens have been (illegally) deported – migrantised in practice. Typically these deported citizens share characteristics with people who are recognised as vulnerable to signing false confessions: Black, with poor literacy and mental health challenges (2019, p. 9).

We would argue, however, that recognising the different ways in which the term ‘migrantisation’ is used brings further clarity to this endeavour, whilst allowing inter-connections to be brought out more explicitly. For some, ‘categorical migrantisation’ leads to ‘migrantisation in practice’ as they are forced to relocate across borders, as in Anderson’s example of deportation. In our current research, the ‘experiential migrantisation’ of British citizens attempting to bring an EU partner to the UK can also lead to ‘migrantisation in practice’ for those forced to move to their partner’s country to be reunited, who then experience ‘categorical migrantisation’ in their new country of residence.

Currently, scholarship on what we have termed ‘categorical migrantisation’ is particularly active, producing valuable insights and empirical exploration. We have suggested that it is useful to conceptualise categorical migrantisation as sets of processes occurring in a variety of domains and at multiple levels, in ways which may or may not coincide or interact. These processes are contextual (both spatial and temporal), and intersect with other dynamics of power – in particular racialisation and class. This model of categorical migrantisation provides a further basis for systemising research in this area, ensuring that research design includes the questions of what domains, levels of analysis, context and intersecting positionalities are to be included, and to draw attention to relationships with wider dynamics.

Our new terminology to distinguish between usages, and the more systematic approach to migrantisation it suggests, could also bring apparently separate literatures into dialogue. Whilst the ‘demographic’ usage has developed separately from the ‘categorical’ and ‘analytical perspective’ approaches, the identification of a population as ‘demographically migrantised’ rests precisely on the categorisation of some people as ‘migrants’ in the domains of data, policy and academic discourse – creating space for reflection on the implications of uncritical usage of such classification, and the potential for ‘demigranticising’ this area of research. Two recent publications demonstrate the potential of this cross-fertilisiaton. Amelina and colleagues (2024) consider the implication of the Covid pandemic for a care sector dependent on ‘migranticized’ workers, whilst Maâ (2024) goes further in her nuanced study of the migrantisation of the borderwork labour force in North Africa, showing how ‘peer intermediaries’ employed to encourage voluntary returns are produced as othered and homogenised ‘migrants’ naturally able to work with other ‘migrants’, whilst simultaneously offered opportunities differentiating them from their ‘peers’. This kind of work, bringing empirical findings of ‘demographic migrantisation’ together with the more critical insights from the study of ‘categorical migrantisation’ has the potential to unite these previously separate intellectual lineages in the study of migrantisation.

We end with one further consideration. The term ‘migrantisation’ remains rather ‘ugly’ and ‘inelegant’ (Dahinden & Anderson, 2021), but does important work in the reflexive, anti-essentialist turn in Migration Studies. We have therefore explored its various usages and suggested ways to enhance the clarity of discussions in which it is employed. However, whilst as migration scholars we find ‘migrantisation’ a more intuitive term than ‘doing migration’ (a banner under which closely related and highly useful conversations are taking place), we end with one remaining concern: its appeal to non-specialist audiences. We would welcome a discussion on whether the term ‘migrantisation’ will constitute an effective tool of communication with policy makers, funders, and academic colleagues from other specialisms. If not, we may have to consider the possibility of developing alternative vocabularies to communicate the important ideas contained in ‘migrantisation’ scholarship to non-specialist and non-academic audiences.

## Data Availability

Our interview data cannot be shared openly, to protect participant privacy. Anonymised research data from the Brexit Couples project will be archived with the UK Data Service after the completion of the project in 2026.

## Footnotes

1 We use the UK English ‘-ise’ spelling throughout, but ‘-ize’ is also used elsewhere in the literature.

2 See Murji & Solomos, 2005 for discussion on the complexity of racialisation.

3 All references to Anderson are to Bridget Anderson unless otherwise specified.

4 Brexit Couples Project (https://brexitcouples.ac.uk/)

5 Limitations of Google Scholar for review have, for example, been explored with regard to medical research: Gusenbauer & Haddaway, 2020.

6 Iosofides (2017) argued instead for a critical realist approach.

7 We write in the aftermath of the Summer 2024 riots in the UK, when rioters set fires at hotels they understood as accommodating ‘migrants’.

8 The slogan “we didn’t cross the border, the border crossed us” emerged in Mexican-American migrant rights movements. For an example in Europe, see e.g., Sardelić (2018).

9 Some scholars have taken issue with this suggestion, on the grounds that the constructs of ‘migrants’ and ‘migration’ are too important not to take centre stage in analysis (Anthias, 2023).

10 Including through notions of ‘homeland’ or ‘return’ (Amelina, 2020).
